# Supplementary material for: In Vitro Anticancer Activity of Methanolic Extract of Justicia adhatoda Leaves with Special Emphasis on Human Breast Cancer Cell Line
Source: Molecules. 2022 Nov 25;27(23):8222. doi: 10.3390/molecules27238222 (PMC9737760; doi:10.3390/molecules27238222)
Supplement: Supplementary file 1 [file molecules-27-08222-s001.zip › molecules-1950859-supplementary.pdf]

Supplementary file

Supplementary Table S1: List of phytochemicals obtained by LC-MS analysis of *J. adhatoda* methanolic extract.

Sl no	Name	Formula	m/z	RT	Width	Height	Area
1	**Alpha d galactopyranose**	C6H12O6	203.0512	3.69	0.26	25367	397353
2	**Vasicinol**	C11H12N2O2	205.0966	5.35	0.26	608518	9994763
3	**Vasicine**	C11H12N2O	189.1017	7.07	0.48	5392962	197106506
4	**Vasicinolone**	C11H10N2O3	219.0759	8.59	0.29	420243	7716009
5	**Resveratrol 5 O-glucoside**	C20H22O8	391.1392	8.79	0.22	23854	323315
6	**Sinapine**	C16H24NO5	311.1711	8.98	0.2	3520	29381
7	**Resveratrol 5 O-glucoside**	C20H22O8	391.1385	9.3	0.45	8531	197992
8	**Adhavasinone**	C12H14N2O2	219.1122	9.34	0.32	1479562	29031855
9	**d-Viniferin**	C28H22O6	455.1511	9.89	0.2	2624	27455
10	**Vasicol**	C11H14N2O2	207.1117	10.1	0.2	82013	1164407
11	**Dihydrocaffeic acid**	C9H10O4	205.0486	10.23	0.21	15904	205464
12	**Ferulic acid**	C10H10O4	195.0642	10.28	0.22	14893	201236
13	**Paniculide B**	C15H20O5	281.1387	10.92	0.33	23889	411272
14	**Vasicinone**	C11H10N2O2	203.0809	11.49	0.24	3692676	58709084
15	**Pinosylvin**	C14H12O2	235.0701	12	0.25	5880	79402
16	**5-methoxyvasicinone**	C12H14N2O3	235.1067	12.26	0.33	7446	143398
17	**Skullcapflavone I**	C17H14O6	337.0709	13.22	2.84	1552	10614
18	**Andrographidine C**	C23H24O10	461.1443	14.24	0.27	4511	75925
19	**Deoxyvasicinone**	C11H10N2O	187.0855	14.82	0.24	150137	2318336
20	**Andropanoside**	C26H40O9	497.2717	15.13	0.54	1886	12211
21	**Ferulic acid**	C10H10O4	195.0639	15.88	0.22	9536	130269
22	**d-Viniferin**	C28H22O6	477.1379	16.05	0.26	5618	74795
23	**Andrographidine D**	C25H28O12	477.1379	16.05	0.23	5061	74475
24	**Gardenin A**	C21H22O9	419.134	17.38	0.25	2259	23320
25	**Andrographidine A**	C23H26O10	463.1604	17.39	0.25	11749	185201
26	**14-Deoxy-11-hydroxyandrographolide**	C20H30O5	351.2128	17.4	0.5	2465	14134
27	**3,14-Dideoxyandrographolide**	C20H30O3	319.2258	18.16	0.23	1830	15684
28	**Deoxyandrographiside**	C26H40O8	481.2784	18.18	0.22	10075	140837
29	**19-O-acetylanhydroandrographolide**	C24H34O7	457.2212	18.32	0.17	3406	18481
30	**Andropaniculosin A**	C18H16O7	345.097	19.02	0.28	2839	38387
31	**Homoveratric acid**	C10H12O4	194.1509	19.78	0.18	4182	49807
32	**12-Hydroxyandrographolide**	C19H28O7	391.1752	20.25	0.23	29753	425541
33	**14-Deoxy-11-hydroxyandrographolide**	C20H30O5	351.2129	20.54	0.53	21040	603543
34	**Methoxyphenylacetic acid**	C9H10O3	167.0693	20.73	0.4	7156	128553
35	**5-hydroxy-7,8,4’-trimethoxyflavone**	C18H16O6	329.1018	20.93	0.27	5444	99737
36	**5-Hydroxy-7,8,2',3'-tetramethoxyflavone**	C19H18O7	359.1127	21.25	0.26	18156	350292
37	**8,17-Epoxy-14-deoxyandrographolide**	C20H30O5	353.2286	21.61	0.3	25543	478869
38	**Isoswertisin**	C22H22O10	447.1284	22.72	0.33	2753	46492
39	**Andrographidine C**	C23H24O10	461.1437	22.75	8.3	1379	11271
40	**Andrographolactone**	C20H24O2	297.1831	23	0.16	5444	57374
41	**Stigmastanol ferulate**	C39H60O4	615.4297	23.49	0.51	5781	161224
42	**5-Hydroxy-7,8,2',3'-tetramethoxyflavone**	C19H18O7	359.1115	23.68	1.38	1004	11710
43	**14-deoxy-11,12-didehydroandrographolide**	C20H28O4	333.2021	23.93	0.22	19265	258614
44	**14-deoxy-11,12-didehydroandrographolide**	C20H28O4	355.1868	25.87	0.2	5937	67107
45	**Adipic acid**	C6H10O4	147.0636	26.18	0.19	3023	31977
46	**Andrographic Acid**	C20H28O6	387.1786	26.87	0.17	7561	74839
47	**Piceatannol**	C14H12O4	245.077	27.21	0.23	110784	1869280
48	**Tocopherol**	C29H50O2	453.3666	27.37	0.26	6114	80583
49	**14-acetylandrographolide**	C22H32O6	415.2097	27.39	0.2	21115	294302
50	**tetradecanoic acid/ Myristic acid**	C14H28O2	251.1979	27.75	0.23	5296	27296
51	**Vasnetine**	C19H17N3O3	336.1328	27.87	0.21	257935	3489968
52	**4-Hydroxyphenylacetic acid**	C8H8O3	153.0535	27.88	0.23	19601	262864

53	**5-Hydroxy-7,8,2',5'-tetramethoxyflavone-5-O-β-D-glucopyranoside**	C25H28O12	543.1479	28.1	0.19	3240	28007
54	**14-deoxy-11,12-didehydroandrographolide**	C20H28O4	333.2017	28.28	0.2	4440	42215
55	**8,17-Epoxy-14-deoxyandrographolide**	C20H30O5	375.21	28.44	0.18	2403	16696
56	**Paniculide C**	C15H18O5	279.1205	28.69	0.2	2248	18200
57	**tetradecanoic acid/ Myristic acid**	C14H28O2	251.1991	29.31	0.18	7730	83687
58	**14-deoxy-14,15-dehydroandrographolide**	C20H28O5	319.1865	29.34	0.18	10598	115180
59	**3-O-beta-D-glucopyranosyl-andrographolide**	C26H40O10	535.2561	30.28	0.19	18200	223783
60	**Dehydroandrographoline**	C20H28O5	349.1968	30.4	0.2	26773	347701
61	**Methoxyphenylacetic acid**	C9H10O3	167.0691	30.41	0.24	35228	578309
62	**5,7,8-Trimethoxydihydroflavone**	C18H18O5	337.102	30.94	0.17	4596	45821
63	**Cinnamic acid**	C9H8O2	149.0584	30.94	0.19	5718	55195
64	**Resveratrol**	C14H12O3	229.0857	31.3	19.58	1544	15456
65	**Andrographolic acid**	C20H32O6	391.2134	31.46	0.16	15030	145413
66	**Palmitic acid**	C16H32O2	279.23	31.63	0.12	27229	229798
67	**1,2-Dihydroxy-6,8-dimethoxyxanthone**	C15H12O6	311.0542	32.33	0.25	3487	47362
68	**alpha-Linolenic acid**	C18H30O2	279.2312	33.09	0.23	1047003	15455407
69	**Stigmasta-5,22-dien-3-ol**	C29H48O	413.3729	33.26	0.46	11126	241485
70	**Monostearin**	C21H42O4	359.3148	33.93	0.2	14164	168599
71	**Paniculide A**	C15H20O4	265.1428	34.11	0.2	5168	52919
72	**6'-Acetylneoandrographolide**	C28H44O8	531.2918	34.37	0.18	7960	86793
73	**Deoxyandrographolide**	C20H30O4	335.2174	35.34	1.09	2977	26605
74	**Sitosterol**	C29H50O	437.3724	35.61	0.38	28350	646711
75	**Octadecanoic acid**	C18H36O2	307.2616	36.14	0.17	3911	34236
76	**3-Oxo-14-deoxy-11, 12-didehydroandrographolide**	C31H39N3O6	329.2095	37.85	0.2	3342	30789
77	**14-Deoxyandrographolide**	C20H30O4	337.1992	38.25	0.14	3795	30473
78	**Monostearin**	C21H42O4	359.314	38.32	0.15	7642	69569
79	**Phytol**	C20H40O	319.2977	39.87	0.2	5333	70295
80	**Deoxyandrographolide**	C20H30O4	357.2015	41.34	0.18	2786	19677
81	**Tocopherol**	C29H50O2	431.3869	44.09	1.18	1808	10739

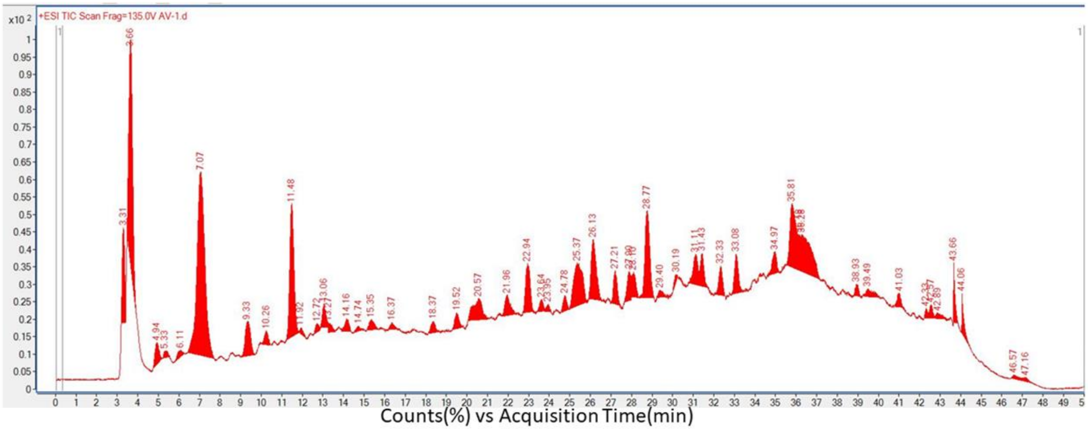

Supplementary Figure S1- LCMS *Justicia adhatoda* leaf extract. The chromatogram was obtained by Q-TOF-LCMS analysis of *Justicia adhatoda* leaf extract to determine its phytoconstituents.

FIGURE 3

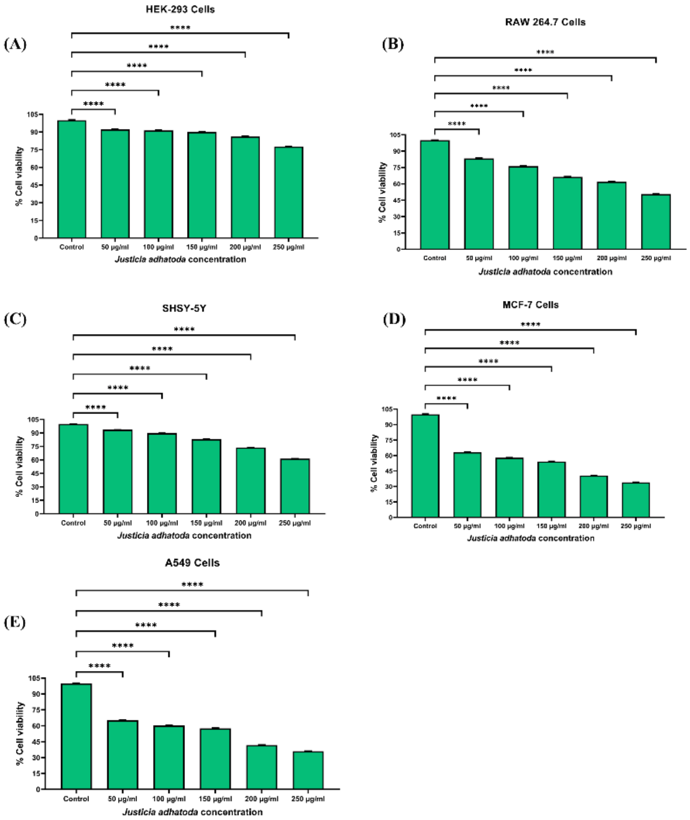

Supplementary Figure S2- MTT assay of *J. adhatoda* leaf extract in different cell lines.

Supplementary Table S2: The gradient of the mobile phase for chromatographic separation

**Time(min)**	**Acetonitrile (%)**	**Methanol (%)**	**0.5% acetic acid in water (%)**
**0-30**	12-25	0	88-75
**30-45**	25-23.5	0-11	75-65.5
**45-68**	23.5-35	11-4	65.5-61
**68-85**	35	4	61
**85-90**	35-50	4-0	61-50
**90-95**	100	0	0
**95-100**	100	0	0
